# Effects of salinity and nutrient stress on a toxic freshwater cyanobacterial community and its associated microbiome: An experimental study

**DOI:** 10.1111/1758-2229.70029

**Published:** 2024-10-24

**Authors:** Océane Reignier, Enora Briand, Fabienne Hervé, Elise Robert, Véronique Savar, Simon Tanniou, Zouher Amzil, Cyril Noël, Myriam Bormans

**Affiliations:** ^1^ IFREMER, PHYTOX, Laboratoire GENALG Nantes France; ^2^ IFREMER, PHYTOX, Laboratoire METALG Nantes France; ^3^ IFREMER, IRSI – Service de Bioinformatique (SeBiMER) Plouzané France; ^4^ UMR CNRS 6553 ECOBIO, University of Rennes Rennes France

## Abstract

We aimed to evaluate the ability of naturally occurring colonies of *Microcystis*, embedded in a thick mucilage, to persist in estuarine waters. In two batch experiments, we examined the dynamics of microbial communities, including cyanobacteria and associated heterotrophic bacteria, sampled from the field during both a cyanobacterial bloom (non‐limiting nutrient condition) and the post‐bloom period (limiting nutrient condition), and subjected them to a salinity gradient representative of the freshwater‐marine continuum. We demonstrated that both *Microcystis aeruginosa* and *M. wesenbergii* survived high salinities due to osmolyte accumulation. Specifically, prolonged exposure to high salinity led to betaine accumulation in the cyanobacterial biomass. The relative abundance of the *mcyB* gene remained around 30%, suggesting no selection for toxic genotypes with salinity or nutrient changes. Microcystins were predominantly intracellular, except at high salinity levels (>15), where more than 50% of the total microcystin concentration was extracellular. In both nutrient conditions, over 70% of the heterotrophic bacterial community belonged to the Gammaproteobacteria family, followed by the Bacteroidota. Bacterial community composition differed in both size fractions, as well as along the salinity gradient over time. Finally, genus‐specific core microbiomes were identified and conserved even under highly stressful conditions, suggesting interactions that support community stability and resilience.

## Graphical abstract

This study demonstrated the strong halotolerance of natural *Microcystis* colonies, which was associated with the production of osmolytes. Bacterial community composition was primarily structured by salinity, followed by time, and differed between the attached and free fractions. The identified attached core microbiomes were genus‐specific and conserved across the salinity gradient, even under highly stressful conditions. These results suggest interactions within the cyanosphere that support community stability and resilience.

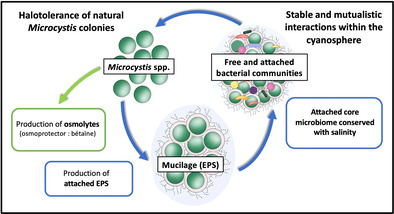

## INTRODUCTION

The contamination of freshwater and estuarine waters by toxic cyanobacteria, predominantly *Microcystis*, is extensively documented worldwide (Bormans et al., 2019; Preece et al., 2017) as a response to both anthropogenic activities and climate change (O'Neil et al., 2012). This emerging challenge is likely to intensify, as indicated by various studies (De Souza et al., 2018; Lehman, 2022; Peacock et al., 2018). The intensification is further exacerbated by hydrologic modifications, changes in land use, drought, and rising sea levels (Huisman et al., 2018; Nielsen et al., 2003; Verspagen et al., 2006; White & Kaplan, 2017), resulting in significant ecological, health and economic risks (Buratti et al., 2017; Quiblier et al., 2013). However, research on the transfer of natural *Microcystis* blooms and their toxins across the freshwater‐marine continuum has been limited. A recent study by Reignier et al. (2024) was the first to report on the selection of potentially microcystin (MC)‐producing *Microcystis* within a *Microcystis* community along a salinity gradient together with an increase in cellular MC quota. Additionally, Bormans et al. (2019) and Réveillon et al. (2024) documented the cell lysis and release of dissolved MCs in surrounding waters at high salinities. Despite these advances, more studies are needed to confidently assess genotype selection based on environmental conditions, considering the potential genetic variability within colonies, blooms, and over time and space (Briand et al., 2009; Sabart et al., 2010; Yancey et al., 2022). Moreover, understanding the intracellular regulation of toxin production is crucial for addressing potential ecological and health risks (Bashir et al., 2023).

Most of the experimental studies conducted on the impact of salinity on *Microcystis* have revealed that these toxic freshwater cyanobacteria can withstand mesohaline conditions (salinities up to S = 18 PSU) (Georges des Aulnois et al., 2019). This resilience is attributed to both inter‐ and intraspecific variations in the ability of some *Microcystis* species/strains to acclimatize to increasing salinity gradients (Georges des Aulnois et al., 2019; Orr et al., 2004; Tonk et al., 2007). This halotolerance of specific strains of *Microcystis* is thought to be associated, in part, with the presence of certain genes involved in the production of compatible solutes, such as sucrose and trehalose. These osmolytes have a crucial role in balancing intracellular and extracellular osmotic pressure (Georges des Aulnois et al., 2019, 2020; Hagemann, 2011; Sandrini et al., 2015; Tanabe et al., 2018). However, it is important to note that these findings are derived from laboratory‐based experiments carried out on single‐cell and monoclonal strains, typically in nutrient rich media. This leaves a critical gap in our understanding regarding the response of natural *Microcystis* colonies, which may exhibit distinct physiological status, to an increase in salinity. Recently, in situ monitoring of natural populations of *Microcystis* along a freshwater‐marine continuum revealed extensive accumulations of trehalose and betaine with an increasing salinity gradient (Reignier et al., 2024). This highlights a more complex internal defence strategy adopted by natural *Microcystis* colonies in response to salinity stress. Further exploration of this strategy is essential, particularly in consideration of various environmental conditions.

In addition, in their natural habitat, *Microcystis* forms large colonies surrounded by a thick mucilage which is thought to act as an external defence strategy in response to environmental stresses (Kehr & Dittmann, 2015). Recent studies have highlighted its potential protective role against osmotic stress (Reignier et al., 2023), allowing colonies of *Microcystis* to endure and thrive even in salinities above 20 (Bormans et al., 2023; Reignier et al., 2023; Xiao et al., 2019).

Furthermore, the formation of colonies provides a distinct habitat from the surrounding water, referred to as the cyanosphere (Bell & Mitchell, 1972). This cyanosphere serves as a habitat for heterotrophic bacteria embedded in the mucilage (Parveen et al., 2013), forming the attached *Microcystis* microbiome. This microbiome may play an important role in cyanobacterial bloom development (Cook et al., 2020; Garcia et al., 2015). Therefore, the success of *Microcystis* in adapting to changing environments may be the cooperative microbial interactions within the cyanosphere (Cook et al., 2020; Pound et al., 2021). Recently, Reignier et al. (2024) demonstrated that salinity played a crucial role in shaping the heterotrophic bacterial community associated with a *Microcystis*‐dominated bloom during its 2‐day transfer along a freshwater‐marine continuum. The research also revealed that the mucilage‐associated microbiome was conserved along the continuum, even under different nutrient conditions, suggesting a strong interaction between *Microcystis* and its microbiome and a likely protecting role of the mucilage against an osmotic shock.

As a result, we aimed to evaluate the ability of naturally occurring toxic colonies of the *Microcystis* genus to persist in estuarine waters. We investigated the genetic, physiological, and metabolic processes within the cyanosphere that contribute to the response to an increase in salinity under both non‐limiting and limiting nutrients conditions. Through two batch experiments conducted over a period of 6–9 days, we examined the dynamics of microbial communities (cyanobacteria and associated heterotrophic bacteria) sampled from the field and subjected to controlled conditions representative of the land‐sea continuum during both a cyanobacterial bloom and post‐bloom period. We hypothesized that (1) *Microcystis* would dominate the cyanobacterial community at high salinity with higher biomass in nutrient replete conditions, (2) salinity combined with nutrient limitation would select toxic genotypes, (3) osmolytes profiles would differ with the salinity gradient, and (4) salinity would structure the cyanobacterial microbiomes with genus‐specific conserved core microbiomes.

## EXPERIMENTAL PROCEDURES

### Experimental setup

The natural *Microcystis* colonies were sampled during two sampling campaigns, corresponding to a phytoplankton bloom (September 6, 2021) and a post‐bloom (September 21, 2021) in the Pen Mur freshwater reservoir (47°33′45.0288″ N, 2°29′31.667″ W), located upstream to the Pen Lan estuary (Brittany, France). Regularly monitored by the Regional Health Agency, this site was chosen due to the recurrent proliferation of cyanobacteria of the genus *Microcystis* (Bormans et al., 2019; 2020). For both dates, 10 L of Pen Mur's surface water (depth 0.25 m) were filtered on a 500 μm net, and then brought directly to the laboratory in the dark conditions at 4°C. We deliberately allowed the water sample to stand for a few hours to enable phytoplankton cells, such as diatoms, to settle and cyanobacteria to form a scum on the surface. This scum constituted our inocula, referred to here as the bloom and post‐bloom inoculum.

In the first experiment, called “Nutrient+,” we inoculated the non‐limiting nutrient bloom inoculum in 2 L of modified BG11 medium (Rippka et al., 1979) to maintain a non‐limiting nutrient condition during 6–9 days experiment. Salinities were adjusted to S = 0, 5, 10, 20, and 25 PSU using artificial sea salt (Instant Ocean sea salt, Aquarium Systems). Salinity was checked using a conductivity meter Cond 3110 Set 1 (WTW, Oberbayern, Germany).

In the second experiment, called “Nutrient−,” the nutrient limited post‐bloom inoculum was added in 2 L of Pen Mur reservoir water filtered through 0.2 μm without addition of nutrient to test the combined effect of salinity and nutrient limitation. To ensure sufficient growth during the experiment, we chose not to test the highest salinity S = 25 PSU and to use an intermediate salinity, salinity S = 15 PSU, in addition to the four salinities tested in Nutrient+ condition (i.e., S = 0, 5, 10, and 20 PSU). Indeed, as the physiological state (through the photosynthetic activity not shown) of the first community (Nutrient+ condition) showed that the cyanobacteria were dying at S = 25 PSU we reduced the value to S = 20 PSU as we also added a second stress (Nutrient− condition). For salinities, ≥15 PSU both experiments were stopped after 6 days.

For both batch experiments, the volume of the inoculum was adjusted to obtain an initial *Microcystis* cell concentration of 10^5^ cells ml^−1^. All treatments were run in triplicates in 4 L Erlenmeyer flasks placed in an incubation chamber maintained at constant temperature of 23°C under a 12:12 h light: dark cycle using cool‐white fluorescent tubes (Toshiba, 15 W, FL15D) with an illumination of 30 μmol photons m^−2^ s^−1^. Each flask was gently manually shaken twice a day, and their position in the incubation chamber was randomly changed every day. Samples were harvested on days 2, 6, and 9 for salinities S = 0, 5, and 10 PSU and on days 2, 4, and 6 for salinities S = 15, 20, and 25 PSU for chemical and molecular analyses. Samples were taken every 2 days for phytoplankton identification and counting.

### Nutrient concentrations

Dissolved nutrient concentrations (phosphate and nitrate) were measured at the beginning, middle and at the end of each experiment from a sampling volume of 150 ml filtered on GF/F filters (Whatman). Phosphate was measured with the method of Murphy and Riley (1962) and nitrate was measured after reduction to nitrite on a cadmium‐copper column (Henriksen & Selmer‐Olsen, 1970) both using a sequential Gallery analyser (Thermo Fisher).

In the Nutrient+ experiment, nitrate and phosphate concentrations were non‐limiting to phytoplankton growth at all salinities until the end of the experiment (N‐NO_3_ concentration ranged from 16 to 20 mg L^−1^ and P‐PO_4_ from 2.6 to 4.5 mg L^−1^).

In the Nutrient− experiment, nitrate concentration in the inoculum was N‐NO_3_ = 1.7 mg L^−1^ and remained relatively low for each salinity until reaching a minimum at the end of the experiment (N‐NO_3_ concentration ranged from 0.8 to 1.2 mg L^−1^). Phosphate concentrations were limiting and below the limit of quantification (P‐PO_4_ < 0.01 mg L^−1^) in all samples from the start of the experiment.

### Phytoplankton diversity

Water sample aliquots (2 ml) were fixed with acidic Lugol's solution (1% final concentration) and stored at 4°C in the dark until analysis. Identification and counting were performed at a magnification of ×320 with an inverted microscope (Zeiss Axio observer 5) according to the standard protocol for application of the Water Framework Directive (Version 3.3.1) (Laplace‐Treyture et al., 2009). Species determination was based on morphological criteria using reference books (Bourelly, 1985; Komárek & Anagnostidis, 2008), and phytoplanktonic biovolumes were based on the geometrical estimation suggested by Sampognaro et al. (2020) for *Microcystis* species and Sun and Liu (2003) for the other taxa. Finally, a two‐way ANOVA was performed to evaluate the significance of the batch, time and salinity effect and the coupled effect of these variables on the cell volume of *M. aeruginosa* and *M. wesenbergii*.

### Chemical analysis of cyanotoxins and osmolytes

#### Cyanotoxin analyses

As described in Bormans et al. (2019), 25 ml was filtered through a 1.2 μm GF/C glass filter (Whatman) to separate the cell pellet for the intracellular toxins and the filtrate for dissolved extracellular toxins. Both filters and filtrates were frozen at −20°C until chemical analysis.

For intracellular cyanotoxins extraction, filters were ground with 500 mg of glass beads (0.15–0.25 mm; VWR) and 4 ml of MeOH using a mixer mill (MM400; Retsch) for 30 min at 30 Hz. After centrifugation at 13,000*g* for 5 min at 4°C, 500 μl of supernatant were filtered through a 0.2‐μm filter (Nanosep MF; Pall) and frozen until LC–MS/MS analysis. For extracellular cyanotoxins extraction, filtrates were purified on a BondElut C18 SPE cartridge (Solid Phase Extraction; 200 mg—Agilent) according to the ISO 20179 standard method (ISO, 2005). Methanolic extracts (V_Final_ = 4 ml) were also filtered through a 0.2‐μm filter (Nanosep MF; Pall) and then frozen until LC–MS/MS analysis.

LC–MS/MS analysis was performed by Ultra‐Fast Liquid Chromatography (model UFLC, Nexera, Shimadzu) coupled to a triple‐quadrupole mass spectrometer (5500 QTrap; ABSciex) as described in Reignier et al. (2024).

#### Osmolytes analyses

Methanolic extracts prepared for toxin analysis (intracellular) were used for osmolytes analyses (sucrose, trehalose, DMSP, betaine, methionine and proline). LC–MS/MS analysis was performed on a UFLC (model UFLC, Shimadzu) coupled to a triple‐quadrupole mass spectrometer (4000 QTrap, ABSciex) equipped with a turboV ESI source as described in Reignier et al. (2024).

### Analysis of the bacterial community

#### DNA extraction

Duplicates of water samples (100 ml) were sequentially filtered through a 20 μm polycarbonate membrane filter (Whatman) to collect attached‐living bacterial communities and then, the filtrate was filtered through a 0.22 μm polycarbonate membrane filter (Whatman) to recover free‐living bacterial communities. Filters were stored at −80°C prior to DNA extraction. Genomic DNA from 20 to 0.22 μm polycarbonate filters was extracted using the NucleoSpin Plant II DNA extraction kit (Macherey Nagel), as described in Reignier et al. (2024). For each extract (final volume of 100 μl of elution buffer), the DNA purity and concentration were quantified by UV spectrometry (Nanodrop 2000; Thermo Scientific), and then normalized to 10 ng μl^−1^.

#### Quantification of potentially MC‐producing Microcystis cells

The proportion of *mcy* genotypes in the *Microcystis* population was determined by a real‐time PCR analysis using two target gene regions located on the chromosome (Neilan et al., 1995; Tillett et al., 2000): the intergenic spacer region within the phycocyanin (*PC*) operon and the *mcyB* region, which carries out one step in MC biosynthesis encoding for the nonribosomal peptide synthetases adenylation domain that is responsible for the recognition of one variable amino acid of the MC molecule (Mikalsen et al., 2003). The primers and probes used for the *PC* and *mcyB* genes are specific for *Microcystis* (Kurmayer & Kutzenberger, 2003), and have been described previously (Briand et al., 2009).

For each target gene quantification, the real‐time PCR reactions were conducted in duplicates using a CFX Opus Real‐Time PCR system (BioRad) following Reignier et al. (2024). For the two genes, the number of gene copies per sample was calculated using the standard curve of the target gene copy number versus the threshold cycle (Ct) for each fraction (Reignier et al., 2024). The total number of gene copies per sample was obtained by summing the number of gene copies per fraction (attached‐ and free‐living bacterial community fractions).

#### Identification of the microbial consortium by 16S amplicon sequencing

The bacterial community was examined with primers targeting the V4‐V5 hypervariable region of the 16S rRNA gene using universal primers (515F/926R, Parada et al., 2016) assembled with the Illumina adapters. Primers and PCR reaction are described in Reignier et al. (2024). The triplicate PCR products for each sample were pooled before sequencing. Secondary PCR amplification for the addition of the Illumina compatible sequencing adapters and unique per‐sample indexes was conducted at GeT‐PlaGe France Genomics sequencing platform (Toulouse, France). Barcoded amplicons were quantified, quality‐checked, normalized, pooled, and sequenced within one sequencing run using the 2 × 250 paired‐end method on an Illumina MiSeq instrument with a MiSeq Reagent Kit V3 chemistry (Illumina), according to the manufacturer's recommendations. The sequencing dataset was deposited in the European Nucleotide Archive (ENA) under the project number PRJEB70923.

#### Statistical and bioinformatic analyses

For each nutrient condition, cell volumes differences between salinities and over time were tested with two‐ways ANOVAs for both *M. aeruginosa* and *M. wesenbergii*.

Bioinformatic analyses of the raw sequenced data were performed using SAMBA (https://gitlab.ifremer.fr/bioinfo/workflows/samba; v4.0.0), a standardized and automatized metabarcoding workflow developed by the Ifremer Bioinformatics Platform (SeBiMER) with the same parameters as described in Reignier et al. (2024).

The generated ASVs were then taxonomically assigned using a Naive Bayesian method against the SILVA v138.1 database (Glöckner et al., 2017; Quast et al., 2013). Statistical analyses of diversity were carried out on normalized data by applying the CSS method on the R phyloseq object generated by the SAMBA workflow. The alpha diversity was investigated using four indices: Chao1, Shannon, Simpson's inverse, and Pielou. *p*‐Values were calculated to compare alpha diversities based on a two‐sample *t* test using a nonparametric method with Benjamini–Hochberg correction method. To visualize differences in bacterial community composition among salinity conditions, fractions (attached or free) and times, beta diversity analyses were achieved by ordination method using nonmetric multidimensional scaling (NMDS) with Bray–Curtis dissimilarity matrices (Lozupone & Knight, 2005). To identify and quantify the core microbiome, that is, the bacterial taxa shared among all salinity conditions and times in the attached fraction (and in all replicates) as defined by Neu et al. (2021), UpSetR package was used (Conway et al., 2017). All codes and associated results data are available on gitlab (https://gitlab.com/oreignie/DEMISEL_batch.git).

## RESULTS

### Phytoplankton biomass and community composition

**FIGURE 1:**
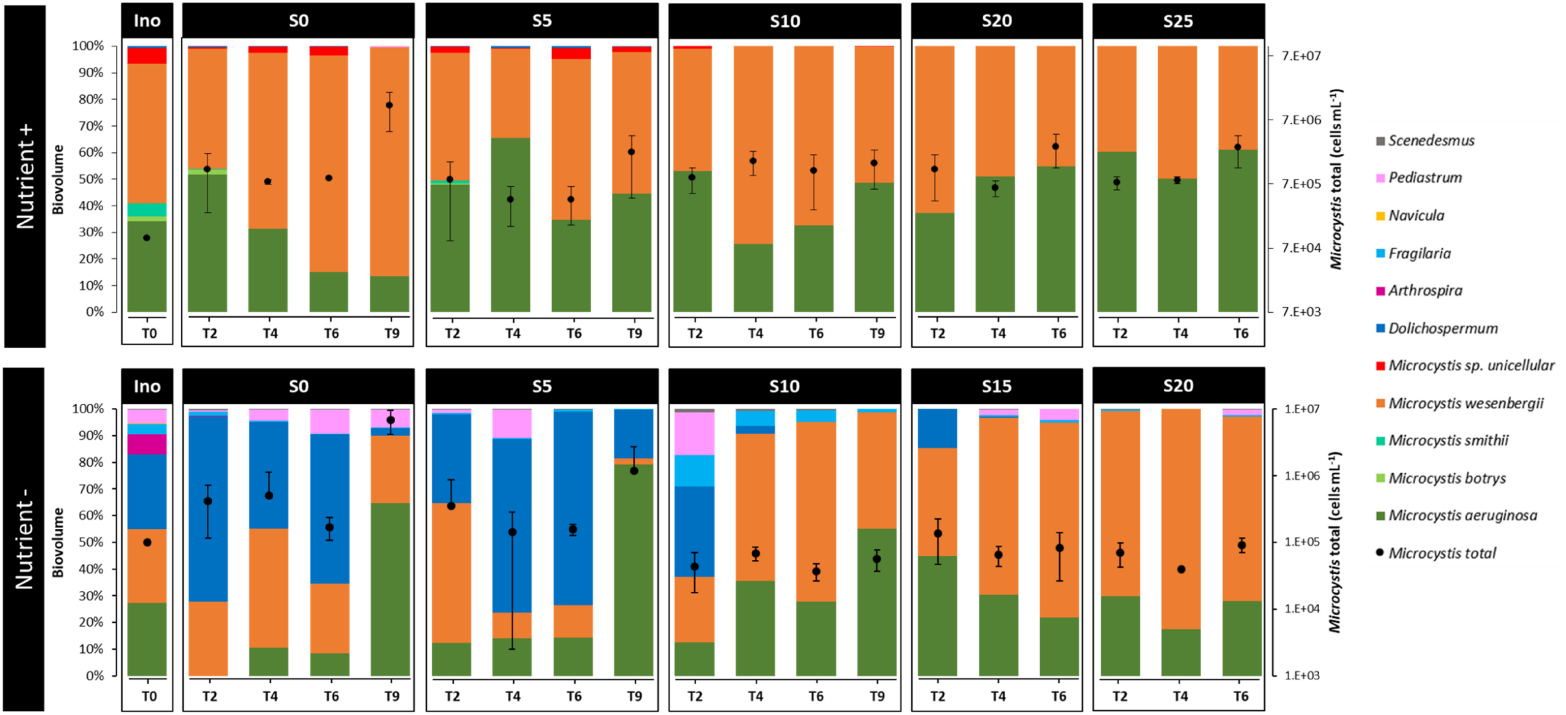
Dynamics of phytoplankton community composition (histograms) expressed as biovolume proportions (see Table S1 for more details) and of total *Microcystis* concentration (cells ml^−1^) as a function of salinity (S0, S5, S10, S15, S20, S25) over time (T0, T2, T4, T6, T9) in Nutrient+ and Nutrient− conditions.

In the Nutrient+ experiment, the phytoplankton community was strongly dominated by cyanobacteria (over 99% in biovolume; Figure 1). The inoculum was composed of more than 50% of *M. wesenbergii*, 30% of *M. aeruginosa*, 7% of unicellular *Microcystis* sp., 5% of *M. smithii*, and 2% of *M. botrys*, for a total *Microcystis* cell concentration of 10^5^ cells ml^−1^. In particular, the cyanobacterial biomass was dominated during the whole experiment by two species of *Microcystis*: *M. aeruginosa* and *M. wesenbergii* in all samples. For each salinity, the total *Microcystis* cell concentration increased slightly to reach a maximum concentration at the final time (1.2 × 10^7^ cells ml^−1^ in S0, 2.2 × 10^6^ cells ml^−1^ in S5, 1.5 × 10^6^ cells ml^−1^ in S10 at T9 and 2.7 × 10^6^ cells ml^−1^ in S20, 2.6 × 10^6^ cells ml^−1^ in S25 at T6; Table S1). After 2 days of experimentation, *M. aeruginosa* and *M. wesenbergii* co‐dominated the total phytoplankton biovolume by approximately 50/50 regardless the salinity condition. At the final time, *M. wesenbergii* was the dominant species accounting for more ~85% of the biovolume in S0 (i.e., 10^7^ cells ml^−1^ at T9), while the cyanobacterial community remained co‐dominated in biovolume by *M. aeruginosa* and *M. wesenbergii* in the other salinity conditions.

In the Nutrient− experiment, the phytoplankton community biomass was also strongly dominated by cyanobacteria (over 70% in biovolume; Figure 1) at all salinity conditions over time. The initial cyanobacterial community biomass (inoculum) was equally composed in biovolume of *M. aeruginosa*, *M. wesenbergii*, and *Dolichospermum*. In S0 and S5, the cyanobacterial community biomass stayed dominated by *Dolichospermum*, *M. aeruginosa* and *M. wesenbergii* accounting for at least 90% of the biovolume over time, except at the final time (T9) where *M. aeruginosa* became the dominant species, accounting for more than 65 and 80% of the total phytoplankton biovolume, respectively in S0 and S5. In parallel, the total *Microcystis* cell concentration initially equal to 10^5^ cells ml^−1^was relatively stable from T2 to T6, and increased to reach a maximum concentration at the final time (6.9 × 10^6^ cells ml^−1^ in S0, 1.2 × 10^6^ cells ml^−1^ in S5; Table S1). At higher salinities conditions, *Dolichospermum* was only present at T2 in S10 and S15, representing 30 and 15% in biovolume of the total phytoplankton biomass, respectively. After 2 days, the cyanobacterial community was composed of *M. aeruginosa* and *M. wesenbergii* with some changes in their contribution as a function of salinity. While *M. aeruginosa* and *M. wesenbergii* co‐dominated under S10, *M. wesenbergii* dominated under S15 and S20. Under these high salinity conditions, the total *Microcystis* cell concentration was relatively stable throughout the experiment (6.9 × 10^4^ ± 3 × 10^4^ cells ml^−1^).

### *Indicator of* Microcystis *physiology: Cell volume*

**FIGURE 2:**
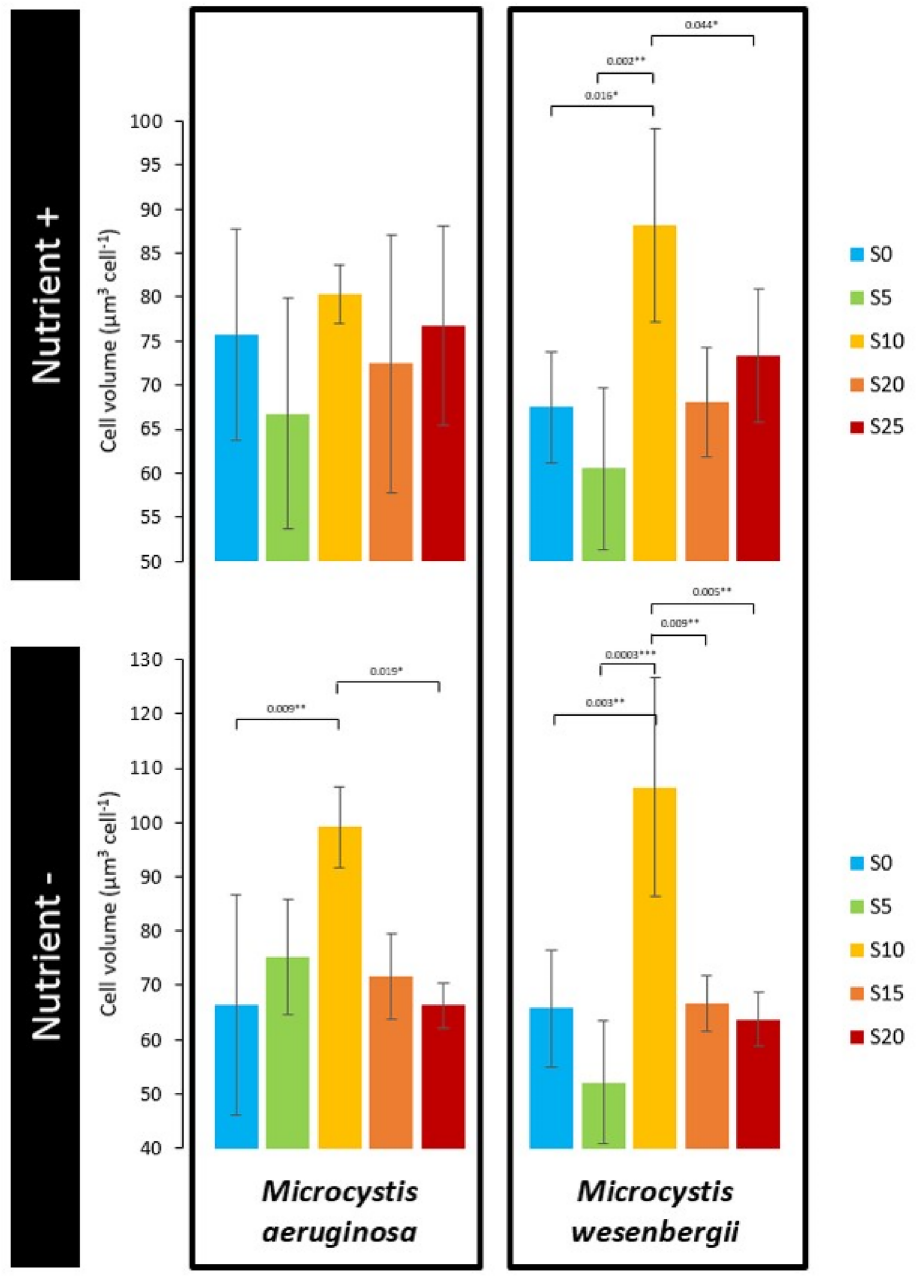
Cell volume of *M. aeruginosa* and *M. wesenbergii* as a function of salinity averaged over time in both Nutrient+ and Nutrient− conditions.

The cell volume of *M. aeruginosa* ranged from 47 to 101 μm^3^ cell^−1^ and *M. wesenbergii* from 36 to 135 μm^3^ cell^−1^ (Figure 2). For both *M. aeruginosa* and *M. wesenbergii*, cell volume was only significantly affected by salinity with no interaction with time or nutrient conditions (two‐ways ANOVAs, *p* < 0.05). Salinity 10 significantly increased the cell volume of *M. aeruginosa* and *M. wesenbergii* compared with other salinities tested under both nutrient conditions (Figure 2).

### Distribution of potentially MC‐producing Microcystis

The dynamics of the potentially MC‐producing *Microcystis* was monitored following the relative abundance of the *mcyB* (marker of the potentially toxic *Microcystis* population) over the *PC* gene (marker of the total *Microcystis* population) (Figure S1). The relative abundance of *mcyB* gene in all salinity conditions was on average 35 ± 10% in Nutrient+ condition and 30 ± 3% in Nutrient− condition. Specifically, the *mcyB* gene's relative abundance increased slightly in low‐salinity environments (S5 and S10) exclusively in Nutrient+, reaching a maximum of 60% of the potentially toxic *Microcystis* population in S5 at T2.

### Intracellular and extracellular MC concentrations

While the total concentrations (intracellular and extracellular) of MCs in the inocula were almost comparable (i.e., 190 and 160 μg L^−1^, respectively in Nutrient+ and Nutrient− experiments; Figure S2), the total concentration of MCs was on average four times higher in all samples in Nutrient+ condition (39 ± 6 μg L^−1^) than in Nutrient− condition (10 ± 4 μg L^−1^) due to the relative increase in biomass.

In both experiments, the concentration of MCs was mostly measured in the intracellular form (Figure S2), except in high salinities (S15, S20, and S25) where the extracellular form accounted for more than 50% of the total MCs concentration measured. No particular selection of intracellular MC variants was observed with the different salinity conditions (Figure S2), but the toxin profile was more diverse in Nutrient+ than in Nutrient− conditions. Seven of the nine MC variants tested could be quantified in the cyanobacterial biomass in Nutrient+ condition, with an average of 50% of MC‐YR, 35% of MC‐LR, 6% of dmMC‐LR, 6% of MC‐RR, and 3% of MC‐LY (and sporadically MC‐LW and MC‐LF), whereas only three variants were quantified in Nutrient− condition with an average of 95% of MC‐LR, 2.5% of dmMC‐LR and 2.5% of MC‐RR. The extracellular toxin profile was similar to the intracellular MC variants in both experiments at high salinities conditions (S15, S20, and S25), while in low salinities (S0, S5, and S10), the dominant variant was MC‐LR in Nutrient+ and MC‐RR in Nutrient− conditions.

### Intracellular osmolytes concentration

**FIGURE 3:**
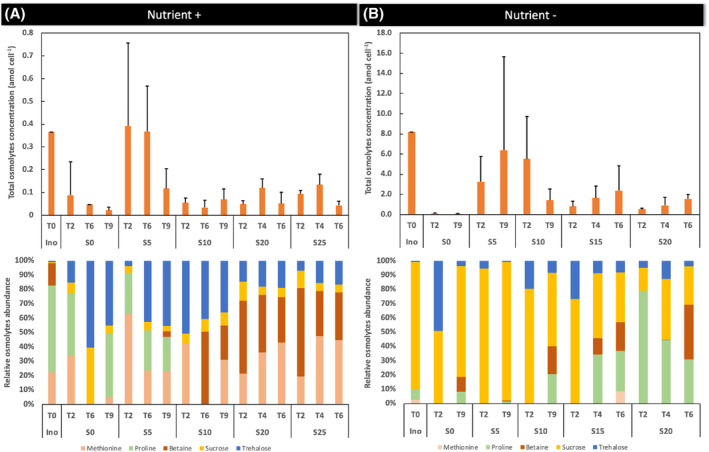
Dynamics of total osmolytes concentration (amol cell^−1^) together with the evolution of the different osmolytes (%) as a function of salinity for both Nutrient+ and Nutrient− conditions.

Total osmolytes concentration was on average 20‐fold higher in Nutrient− than in Nutrient+ condition over time (Figure 3). Regardless of nutrient conditions, the higher total osmolytes concentration was measured at low salinity (mainly in S5).

The osmolytes profile differed between the two experiments, with a distinct inoculation‐compatible solute profile, and was more diverse under Nutrient+ condition over time and salinity than under Nutrient− condition. However, independently of nutrient conditions and inoculum, an exposure to high salinity over time led to betaine accumulation in the cyanobacterial biomass. Among the amino acid family, proline was the dominant osmolyte with increasing salinity and time under Nutrient− condition, while methionine was more stimulated at the expense of the proline under Nutrient+ condition. Among the targeted disaccharides (sucrose and trehalose), the Nutrient+ condition stimulated more trehalose storage, while sucrose was the dominant compatible solute accumulated among all osmolytes screened in Nutrient− condition.

### Structure and composition of the bacterial community

#### Bacterial community composition

**FIGURE 4:**
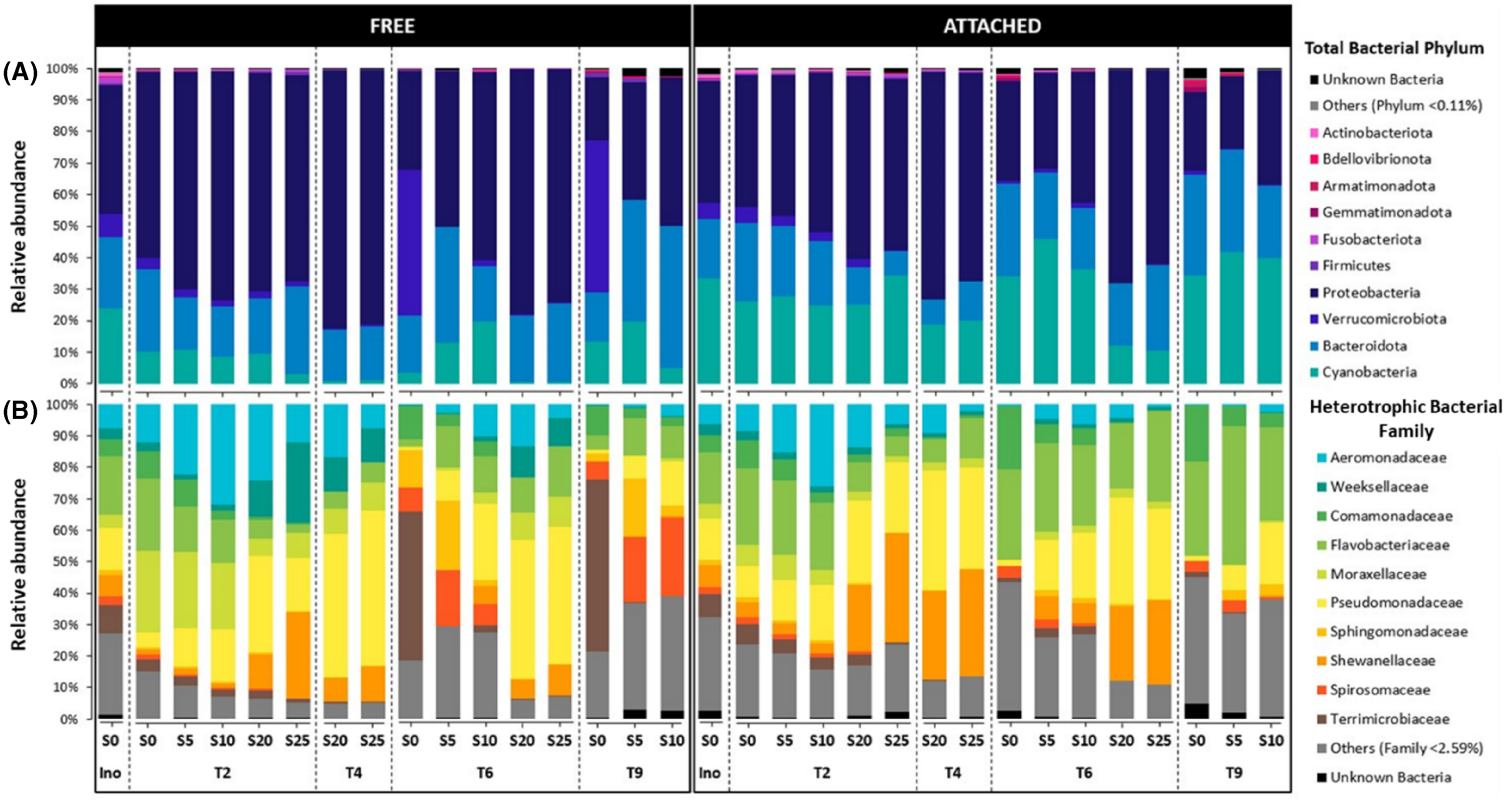
Dynamics of (A) relative abundances of total bacterial sequences at the phylum level in the free‐living (FREE) and attached (ATTACHED) fractions as a function of salinity under Nutrient+ condition. (B) Relative abundances of the heterotrophic bacterial sequences at the family level.

In the Nutrient+ experiment, a total of 3,475,948 sequences, matching 1313 bacterial ASVs, were recovered after quality filtering and removal of Eukaryota, chloroplasts, mitochondrial and unassigned taxa reads. Across samples, most ASVs were assigned to Proteobacteria (52%), Bacteroidota (22%), and Cyanobacteria (79 ASVs accounting for 19% of the total reads) (Figure 4A) with the highest relative abundances of Cyanobacteria found in the attached fraction (30 ± 10%). The cyanobacterial community was dominated by the Microcystaceae family (over 80%) (Figure S3), mainly represented by two *Microcystis* ASVs (dae8d and 9314f) accounting for 64 and 26% of the cyanobacterial community, respectively (data not shown). The Nostocaceae family, mainly represented by *Dolichospermum* ASV (35895), accounted for less than 20% of the cyanobacterial community, except in the free fraction under S20 and S25 conditions, where it reached 70%.

Sequences corresponding to Cyanobacteria were removed from the ASV table to obtain the heterotrophic bacterial community. As a result, 2,816,070 reads clustered into 1234 ASVs. Then, 54% of the reads were sequenced in the free‐living fraction (1,516,395 reads) and 46% in the attached fraction (1,299,675 reads). Among the top 10 families (Figure 4B), representing 79% of the heterotrophic bacterial community, families belonging to Gammaproteobacteria (i.e., Aeromonadaceae, Moraxellaceae, Pseudomonadaceae, and Shewanellaceae) were dominant in the heterotrophic bacterial community (9, 5, 20, 9%, respectively), followed by the Bacteroidota, mostly represented by Flavobacteriaceae (16.5%), Spirosomacea (4%), and Weeksellaceae (3.5%). The other families represented on average less than 2.6% of the relative abundance of the heterotrophic bacterial community.

**FIGURE 5:**
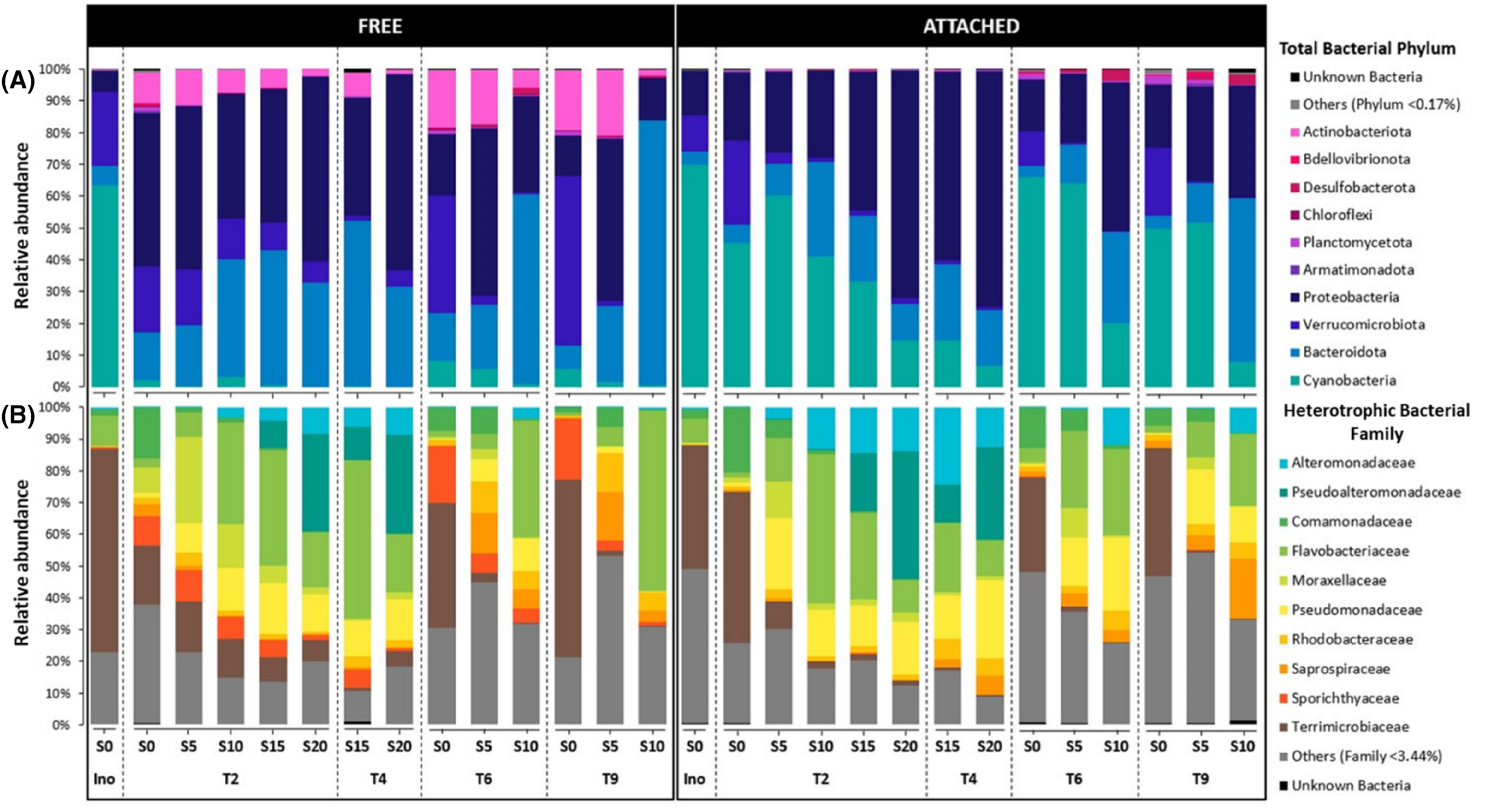
Dynamics of (A) relative abundances of total bacterial sequences at the phylum level in the free‐living (FREE) and attached (ATTACHED) fractions as a function of salinity under Nutrient− condition. (B) Relative abundances of the heterotrophic bacterial sequences at the family level.

Similarly, in the Nutrient− experiment, a total of 3,136,902 high‐quality reads, clustering into 2317 bacterial ASVs, were recovered after quality filtering and removal of Eukaryota, chloroplasts, mitochondrial and unassigned taxa reads. Across samples, most ASVs were assigned to Proteobacteria (37%), Bacteroidota (24%), Cyanobacteria (110 ASVs accounting for 23% of the total reads), Verrucomicrobiota (10%), and Actinobacteriota (5%) (Figure 5A). The highest relative abundances of Cyanobacteria were found in the attached fraction (40 ± 23%). This cyanobacterial community was also dominated by the same two families, the Microcystaceae and the Nostocaceae (Figure S3). The Microcystaceae family was mainly represented by two *Microcystis* ASVs (dae8d and 9314f) accounting for 15 and 5% of the cyanobacterial community, respectively (data not shown). The Nostocaceae family was mainly represented by one *Dolichospermum* ASV (35895) accounting for 75% of the cyanobacterial community. However, the relative abundance of these two families differed according to salinity and time. In S0 and S5, the Nostocaceae family dominated more than 80% the cyanobacterial community over time, while the relative abundance of the Microcystaceae family increased with salinity and over time, reaching a maximum of 85% in S20 at T4.

Sequences corresponding to Cyanobacteria were removed from the ASV table to obtain the heterotrophic bacterial community. As a result, 2,449,248 reads clustered into 2207 ASVs. Then, 60% of the reads were sequenced in the free‐living fraction (1,487,912 reads) and 40% in the attached fraction (961,336 reads). Among the top 10 families (Figure 5B), representing 71% of the heterotrophic bacterial community, families belonging to Gammaproteobacteria (i.e., Alteromonadaceae, Moraxellaceae, Pseudoalteromonadaceae, and Pseudomonadaceae) were dominant in the heterotrophic bacterial community (5, 3, 6, 10%, respectively), followed by the Bacteroidota, mostly represented by Flavobacteriaceae (18%) and Saprospiraceae (3%), and finally by Verrucomicrobiota (Terrimicrobiaceae) accounting for an averaged 14% of the heterotrophic bacterial community. The other families represented on average less than 5% of the relative abundance of the heterotrophic bacterial community.

### Diversity of the heterotrophic bacterial community

In the Nutrient+ experiment, richness (Observed and Chao 1) and alpha diversity (Shannon and Pielou) indices of the heterotrophic bacterial community decreased significantly with increasing salinity (see *p*‐value in Figure S4A). No significant difference was observed between the two fractions for each salinity condition. In the Nutrient− experiment, bacterial communities at S = 5 and above were less rich than the one at S = 0 for each fraction (Figure S4B). No significant difference was observed in the diversity indices, except the attached bacterial community at S = 15, which showed significantly higher diversity.

### Dissimilarities between heterotrophic bacterial communities

**FIGURE 6:**
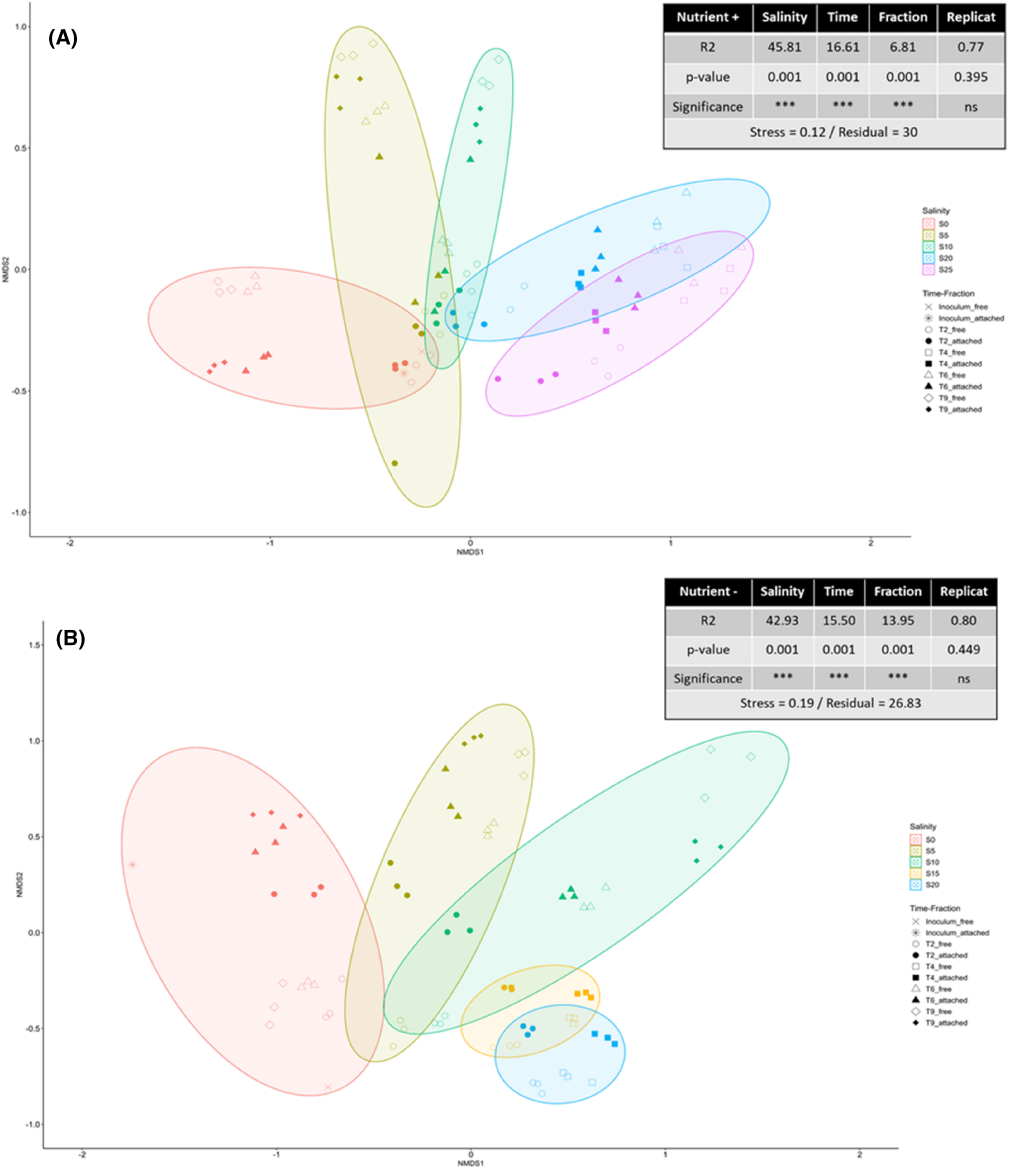
Non‐metric multidimensional scaling (NMDS) ordination, based on Bray–Curtis dissimilarity, of heterotrophic bacterial communities for the Nutrient+ (A) and Nutrient− (B) conditions. The plot axes show NMDS scores. Points in the ordination are coloured by hierarchical clustering assignment. The groups were clustered according to salinity, fraction, time, and replicas.

To investigate dissimilarities between bacterial communities, we performed a NMDS analysis based on Bray–Curtis distance metric (Figure 6A,B). For both Nutrient+ and Nutrient− experiments, NMDS showed that replicas of the same condition consistently clustered together (PERMANOVA *R* ^2^ = 0.77 and 0.80, *p*‐value = 0.395 and 0.449, no significant), highlighting the robustness of the analysis. Salinity, time and fraction were all significant structuring factors of the bacterial communities with a stress value of 0.12 (Nutrient+) and 0.19 (Nutrient−). More specifically, for both Nutrient+ and Nutrient− experiments, dissimilarities in the bacterial community composition increased with increasing salinity (PERMANOVA *R* ^2^ = 45.81 and 42.93, *p*‐value = 0.001) over time (PERMANOVA *R* ^2^ = 16.61 and 15.50, *p*‐value = 0.001) for both distinct attached and free fractions (PERMANOVA *R* ^2^ = 6.81 and 13.95, *p*‐value = 0.001).

### Core microbiomes

**FIGURE 7:**
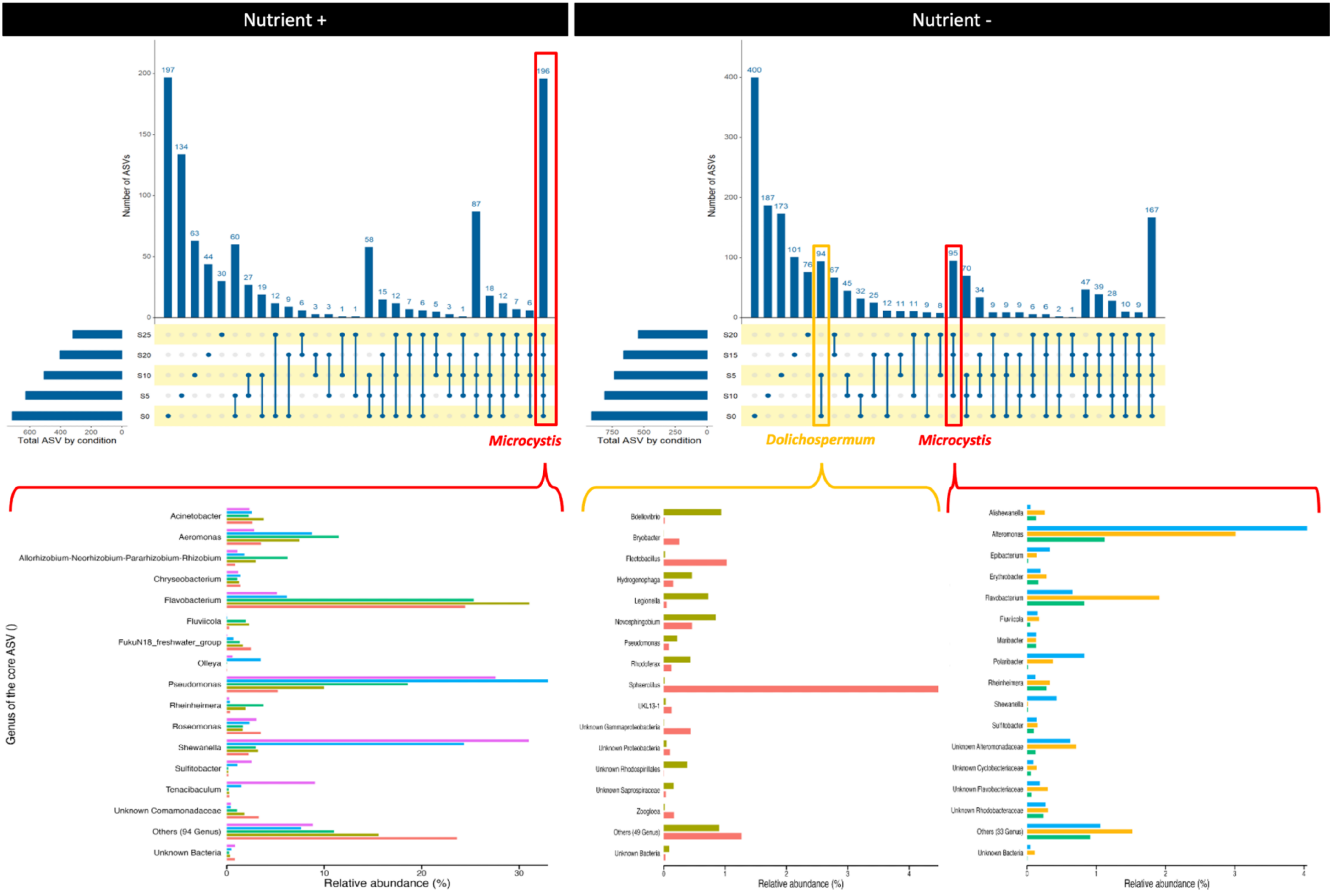
UpSetR plot showing the number of specific or shared ASVs within the attached fraction as a function of salinity for the Nutrient+ and Nutrient− conditions. The bars show the overlap between the indicated samples below. The left bottom panel shows the relative abundances of the main ASVs shared between all salinities under Nutrient+ condition (dominated by *Microcystis*) and the bottom right bottom panel shows the relative abundances of the main ASVs shared between S0 and S5 (dominated by *Dolichospermum*) and between S10, S15, and S20 (dominated by *Microcystis*), respectively, under Nutrient− condition.

To assess whether the mucilage‐associated microbiome was conserved with increasing salinity, we examined the ASVs present in the attached fraction and recovered across all salinity, time, and replicates and defined them as core microbiomes of *Microcystis* for the Nutrient+ experiment. Based on the UpSetR analysis, a total of 196 ASVs (1,151,196 reads) were identified in the Nutrient+ experiment (Figure 7, left). This core microbiome represented 41% of the total heterotrophic bacterial community. Within the attached bacterial community, the relative abundance of this core microbiome varied from 75% in S0 and gradually increased to 97% in S25 (Table S2). Among the core ASVs members, 15 dominant genera (relative abundance >0.93%) were identified and conserved in all salinity conditions (Figure 7, left), such as *Acinetobacter*, *Aeromonas*, *Chryseobacterium*, *Flavobacterium*, *FukuN18_freshwater_group*, *Pseudomonas*, *Roseomonas*, and *Shewanella*.

Similarly for the Nutrient− experiment, based on the UpSetR analysis, we investigated the core microbiomes of *Dolichospermum*‐ or *Microcystis*‐dominated populations, at S0‐S5 or S10‐S15‐S20, respectively (Figure 7, right). A total of 94 ASVs were identified as bacterial taxa shared between S0 and S5. These 94 ASVs represented approximately 5% of the bacterial community attached to *Dolichospermum*. Similarly, between S10, S15 and S20, 95 ASVs were identified as bacterial taxa, representing around 5% of the bacterial community associated with *Microcystis*. Within the shared ASVs members, 15 dominant genera (relative abundance >0.47%) were identified in the attached bacterial community of *Dolichospermum* such as *Bdellovibrio*, *Sphaerotilus*, *Flectobacillus*, *Legionella* and *Novosphingobium*. Similarly, *Alteromonas*, *Flavobacterium*, *Polaribacter*, *Shewanella*, *Fluviicola*, and *Sulfitobacter* were specific dominant genera identified in the attached bacterial community of *Microcystis*.

## DISCUSSION

This study aimed to evaluate the ability of a toxic cyanobacterial community dominated by *Microcystis* to persist in estuarine waters by studying, within the cyanosphere, the genetic, physiological and metabolic processes involved in the response to an increase in salinity and nutrient stress. Hence, the microbial communities (cyanobacteria and associated heterotrophic bacteria), sampled in the field during and after a cyanobacterial bloom, were exposed in Nutrient−enriched and non‐enriched controlled environments to a salinity gradient representative of the land‐sea continuum. We acknowledge that the initial communities (both of cyanobacteria and heterotrophic bacteria) are different for several reasons [nutrients concentrations (N and P), DOC concentrations, cyanophages, etc.]. Nevertheless, here we tested the impact of salinity on both communities, the first one was not nutrient limited during the entire experiment while the second community was nutrient limited during the entire experiment. As both conditions included a zero salinity control, we believe that the role of DOC, cyanophages, and so forth would be included in that control.

### Cyanobacterial community response

The observed strong dominance of cyanobacteria in the phytoplankton community across all salinity conditions in both the Nutrient+ and Nutrient− experiments over time highlighted their range of unique and highly‐adaptable eco‐physiological traits developed over 3.8 billion years (Litchman et al., 2010).

The microscopic observations were in strong agreement with data obtained by 16S rRNA sequencing showing that *Microcystis* ASVs dominated over 80% of the cyanobacterial community except in S0 and S5 of Nutrient− experiment where *Dolichospermum* ASVs dominated more than 80% the cyanobacterial community over time. These results are very promising and suggest a high level of correspondence between taxonomy‐based microscopy and DNA metabarcoding analyses, which is not always observed (Gelis et al., 2024).

In this study, under Nutrient+, *M. aeruginosa* and *M. wesenbergii* co‐dominated the cyanobacterial biovolume over time by approximately 50/50 regardless the salinity condition, while under Nutrient− *M. wesenbergii* dominated in high salinity conditions. These findings are aligned with several previous investigations that have highlighted the relatively high salinity threshold tolerance of *M. aeruginosa* (Lehman et al., 2005; Lewitus et al., 2008; Tonk et al., 2007) and, more recently, *M. wesenbergii* (Reignier et al., 2023, 2024). Both *Microcystis* species have demonstrated an ability to thrive in salinity conditions of up to 20 along a short land‐to‐sea continuum in France (Reignier et al., 2024). This current study builds on these findings by demonstrating that the halotolerance of these two *Microcystis* species extends beyond 6 days, even when they are exposed to nutrient stress conditions, as we had hypothesized. This persistence is most likely attributed to the presence of a robust mucilage layer, which tends to increase in the absence of nutrients (Reignier et al., 2023) and provides physical protection against osmotic shock (Bormans et al., 2023), thereby contributing to the overall ecological resilience of these species. Our results are also in accordance with the laboratory experiment of Li and Li (2012) and the field investigation of Li and Xiao (2016) who analysed environmental factors related to the succession of *M. wesenbergii* and *M. aeruginosa* in freshwater. They reported that in a nutrient favourable environment, *M. aeruginosa* had a stronger competitive ability than *M. wesenbergii*, whereas in a phosphorus‐limited environment, *M. wesenbergii* was more competitive than *M. aeruginosa*. These insights contribute to a better comprehensive understanding of the ecological dynamics and competitive strategies employed by these *Microcystis* species.

In this study under Nutrient− conditions, *Dolichospermum* dominated at low salinities, consistent with other studies suggesting it has a lower salinity threshold than *Microcystis* (Moisander et al., 2002; Tonk et al., 2007). This result aligns with our field study, where *Dolichospermum* was present in the freshwater reservoir but not present in the estuary (Reignier et al., 2024). *Dolichospermum* also showed a considerable advantage under low nutrient conditions, as demonstrated here by the presence of heterocysts (data not shown), indicating its diazotrophic capabilities.

### *Impact of salinity and nutrients on MC‐producing* Microcystis *and MCs*

The dynamics of potentially MC‐producing *Microcystis* in both nutrient conditions were similar with ~30% of the genotypes being toxic. Notably, regardless of nutrient conditions, we observed no selection of potentially MC‐producing *Microcystis* within a *Microcystis* community based on salinity over time. This result indicates that a non‐negligible proportion of toxic genotypes was able to sustain high salinity regardless of nutrient conditions as also suggested by Martínez de la Escalera et al. (2017) and Reignier et al. (2024). However, we did not observe a selection of toxic genotypes with salinity as hypothesized and as did Reignier et al. (2024) in their field study. This suggests the importance of local environmental conditions other than nutrients and salinity on the fitness of potentially MC‐producing and non‐MC‐producing subpopulations as reported within *Microcystis* blooms in different freshwater ecosystems (Briand et al., 2009; Lezcano et al., 2018; Sabart et al., 2010).

The concentration of intracellular MCs was relatively stable over time, irrespective of salinity, within each nutrient experiment. However, intracellular MCs concentrations were higher under non‐limiting nutrient conditions due to higher biomass as was also found by Kramer et al. (2018) in the St Lucie River and Estuary. These results are also in accordance with literature reporting MCs production proportional to growth rate. Indeed, a linear relationship has been observed between MCs production and growth rate in continuous cultures of axenic *Microcystis* under nitrogen‐limited condition (Long et al., 2001; Orr & Jones, 1998), phosphorus‐limited condition (Davis et al., 2010; Oh et al., 2000) or different salinities (Georges des Aulnois et al., 2020).

In both nutrient experiments, the concentration of MCs was mostly measured in the intracellular form, except at high salinity (S15, S20, and S25) where the extracellular form accounted for more than 50% of the total MCs concentration measured. Consistent with literature generally referring to *M. aeruginosa* (Ross et al., 2019), cell lysis at high salinity are likely to occur (Bormans et al., 2019; Martínez de la Escalera et al., 2017). This implies that natural blooms of *Microcystis* might endure during their transfer to estuarine zones with salinities surpassing mesohaline values, potentially resulting in an increase in extracellular MCs.

The MCs profiles of the natural bloom (under both Nutrient+ and Nutrient−) remained similar between salinity conditions, as previously observed with isolated strains (Georges des Aulnois et al., 2019) or natural colonies (Reignier et al., 2024), suggesting that *Microcystis* produced no particular MC analogs in response to salt stress. However, we observed that the MCs profile was more diverse under favourable Nutrient+ than Nutrient−. These changes in variant profiles can be attributed to the selection of cyanobacteria species and strains with the nutrient concentration. A more diversified toxin profile could potentially increase the overall toxicity of cyanobacterial blooms due to the relative toxicity of different MC variants, their ability to persist in the environment, and the sensitivity of organisms exposed to these toxins.

### Physiological adaptation and internal defence strategy

At the single‐cell level, the production and accumulation of compatible solutes is the result of various stress signalling pathways, which are necessary for the survival of the organism in abiotic stress environments by protecting and minimizing stress‐induced oxidative damage, reduced growth and loss of photosynthetic efficiency (Jogawat, 2019). In our study, we observed that the total osmolyte concentrations were on average 20‐fold higher in Nutrient− than in Nutrient+ condition. However, it is noteworthy that the highest total osmolyte concentration was measured at low salinity (S = 5) regardless of nutrient experiment. This observation suggests the existence of a maximum “activation” threshold for this internal strategy, emphasizing that the effectiveness of the osmoregulation mechanism peaks at low salinity levels regardless of nutrient availability. Indeed, some previous studies based on unicellular *Microcysti*s strains indicated that the halotolerance was mediated by the discharge and uptake of water in the cell within few hours after exposure at low salinity thanks to the production and accumulation of compatible solutes (Cantrell et al., 2023; Georges des Aulnois et al., 2019, 2020; Hagemann, 2011; Kirsch et al., 2019). Wang et al. (2022) also found a significant increase in various compatible solutes in natural *Microcystis* colonies in a salt‐water simulation experiment (S = 5 and S = 10), together with an increase in cell size at S = 10. We also observed that at salinity S = 10 the cell volume of *M. aeruginosa* and *M. wesenbergii* significantly increased compared with other salinities tested under both nutrient conditions. This result suggests a maximum turgor pressure threshold reached at S = 10. Tonk et al. (2007), Georges des Aulnois et al. (2019) and Bormans et al. (2023) observed a decrease in cell size at higher salinity (S > 10), suggesting that osmoregulation was exceeded and cells were notwithstanding the high turgor pressure. However, more studies are needed due to variations between strains, timing after exposure, environmental conditions and the composition of the cyanobacterial consortium.

As we hypothesized, osmolytes profiles differed with salinity. We observed a betaine increase at high salinity regardless of nutrient conditions while proline and sucrose increased under Nutrient− condition, and methionine and trehalose were enriched under Nutrient+ condition. Enrichment in trehalose and betaine was also observed during the transfer of a cyanobacterial community dominated by *M. aeruginosa* along a freshwater‐estuary continuum (Reignier et al., 2024). The dominance of *M. wesenbergii* at high salinities under Nutrient− condition could also contribute to the production of distinct osmolytes compared to *M. aeruginosa*. However, further investigations are needed to fully understand this hypothesis. Furthermore, Ataeian et al. (2022) highlighted that more than half of the consortium members of heterotrophic bacteria associated with cyanobacteria were capable of using osmolytes such as sucrose, trehalose, glucosylglycerol, and glycine betaine as an organic carbon source. Genes for glycine betaine biosynthesis were also detected in consortium members affiliated with Proteobacteria and Verrucomicrobiota, phyla frequently observed in association with freshwater cyanobacterial blooms (Li et al., 2020; Reignier et al., 2024; Te et al., 2023; Tromas et al., 2017).

### *Heterotrophic bacterial community associated with* Microcystis

In both nutrient experiments, over 70% of the heterotrophic bacterial community belongs to the Gammaproteobacteria family (i.e., Aeromonadaceae, Alteromonadaceae, Moraxellaceae, Pseudoalteromonadaceae, Pseudomonadaceae, and Shewanellaceae) followed by the Bacteroidota, mostly represented by Flavobacteriaceae. These phyla are those frequently observed within the cyanosphere. Indeed, the Gammaproteobacteria are typically observed in eutrophic conditions (Simonato et al., 2010) and show an increase in abundance from the onset to the decline of cyanobacterial bloom (Parveen et al., 2013), likely due to their copiotrophic metabolism. As the cyanosphere is described as a hotspot for organic matter fluxes (Paerl, 1996; Worm & Søndergaard, 1998), which increase with the salinity (Reignier et al., 2023), this could explain their predominance due to their high efficiency in degrading algae‐derived substrates (Newton et al., 2011; Niemi et al., 2009). In addition, Flavobacteriaceae species exhibit prevalence within the phycosphere of *Microcystis*, as reported by Kim et al. (2020). Note that Actinobacteria, which are usually dominant in freshwater free‐living bacterial community (Humbert et al., 2009; Parveen et al., 2013, Reignier et al., 2024), were in the minority (under Nutrient−) or even almost absent (under Nutrient+) in the free fractions. This can be explained by the fact that the inoculum used for both experiments were sampled in the cyanobacterial scum and were therefore more representative of the bacterial community attached to the cyanobacterial biomass (see the strong similarity for each inoculum between the composition of free and attached bacterial communities; Figures 4 and 5). Nevertheless, the structure of these two bacterial communities (free‐living and attached) has evolved differently over time, with both communities becoming less rich and diverse along the salinity gradient. Our results demonstrated the strongest role of salinity in structuring the cyanobacterial microbiomes as was also observed in our field study during its transfer along a freshwater‐marine continuum for both bloom periods (Reignier et al., 2024). While the richness and diversity of the heterotrophic bacterial community increased along the freshwater‐marine continuum (Reignier et al., 2024), mostly due to the enrichment of estuarine taxa, the experimental study performed under constraining conditions resulted in lower diversity and richness.

Our results underlined the existence of genus‐specific and conserved core microbiomes, suggesting close and potentially beneficial interactions between cyanobacteria and its associated bacteria under varied environmental conditions. Indeed, the core microbiome associated with *Dolichospermum* was different from that associated with *Microcystis* as was also reported by Louati et al. (2016) who showed a lack of bacterial correlation between these genera. Similarly, Lefler et al. (2023) found only one single bacterial taxon (Firmicutes) shared between microbiomes of *Dolichospermum* and *Microcystis* in Lake Okeechobee. This result provides further evidence for specific associations between cyanobacterial species and heterotrophic bacteria (Louati et al., 2016; Pérez‐Carrascal et al., 2021; Zhu et al., 2016). This phenomenon can be attributed either to the different cellular organization (filamentous versus colonial), which may provide distinct habitats for bacteria, or to their contrasting metabolic capacities resulting in different quality and quantity of exudates. Notably, structural and functional differences were found between bacterial communities associated with N_2_‐fixing cyanobacterial strains and those associated with non‐N_2_‐fixing strains (Zhu et al., 2016). Fixed N_2_ by *Dolichospermum* is released as NH_4_^+^, which consequently supports the associated bacterial community (Adam et al., 2016) and prevents them from experiencing nitrogen‐limiting conditions. In addition, *Microcystis* core microbiomes identified in Nutrient+ and Nutrient− were quite similar with the one identified in our field study (Reignier et al., 2024), with *Flavobacterium*, *Shewanella*, and *Fluviicola*, the dominant ASVs in common. Moreover, we observed an overall enrichment of the *Microcystis* core microbiome in response to increased salinity, suggesting a greater resilience of these taxa. Overall, these results highlight the importance of specific interactions between *Microcystis* and its associated bacteria, which are maintained even under highly stressful conditions, likely thanks to the mucilage. Further functional investigations, including metatranscriptomic analyses, would determine which bacteria are actively involved and would contribute to better understand these interactions and their role in the ecological success of *Microcystis*.

## CONCLUSION

These data confirm that *Microcystis* can survive and grow at relatively high salinity for a week, especially when not combined with nutrient limitation. No selection of toxic genotypes with salinity was observed, regardless of nutrient limitation, suggesting more complex relationships with other environmental parameters. Specific osmolytes were produced in response to salinity, likely to alleviate osmotic shock. Salinity strongly structured the associated microbiomes, with genus‐specific core microbiomes conserved. These findings emphasize the importance of understanding the intricate interactions between *Microcystis* colonies and their microbiome, providing valuable insights into their widespread success and adaptive strategies in the face of environmental challenges, particularly salinity fluctuations. These results not only highlight the fate of freshwater cyanobacterial blooms during their transfer to estuaries but also suggest that, in light of freshwater salinization, seen as the principal threat to freshwater ecosystems (Cunillera‐Montcusí et al., 2022), potentially toxic *Microcystis* would likely bloom due to their tolerance to salinity. This would reduce competition with other phytoplankton, alter ecosystem equilibrium, and impact ecosystem health.

## AUTHOR CONTRIBUTIONS

**Océane Reignier:** Writing – original draft; visualization; formal analysis; investigation. **Enora Briand:** Validation; writing – review and editing; supervision; funding acquisition; conceptualization. **Fabienne Hervé:** Investigation; methodology; writing – review and editing. **Elise Robert:** Writing – review and editing; methodology; investigation. **Véronique Savar:** Investigation; methodology; writing – review and editing. **Simon Tanniou:** Investigation; methodology; writing – review and editing. **Zouher Amzil:** Writing – review and editing; supervision. **Cyril Noël:** Writing – review and editing; resources; software. **Myriam Bormans:** Validation; writing – review and editing; supervision; conceptualization.

## CONFLICT OF INTEREST STATEMENT

The authors declare no conflict of interest.

## Supporting information


**FIGURE S1.** Dynamics of the relative abundance (%) of *mcyB* gene over the total *Microcystis* population (*PC* gene) as a function of salinity in both Nutrient+ and Nutrient− conditions.
**FIGURE S2.** Dynamics of intracellular and extracellular microcystins concentrations (μg L^−1^) together with the evolution of the different variants (%) as a function of salinity for both Nutrient+ and Nutrient− conditions.
**FIGURE S3.** Dynamics of relative abundances of cyanobacterial sequences at the family levels in the free‐living (FREE) and attached (ATTACHED) fractions as a function of salinity under Nutrient+ (top panel) and Nutrient− (bottom panel) conditions.
**FIGURE S4.** Alpha diversity box‐plot displaying the number of ASVs observed, Chao1, Shannon and Pielou diversity indices for the free‐living (in blue) and attached (in green) heterotrophic bacterial communities as a function of salinity for the Nutrient+ (**A**) and Nutrient− (**B**) conditions. Solid lines and asterisks indicate a significant difference. P‐values were calculated to compare alpha diversities based on a two‐sample *t* test using a nonparametric method with Benjamini‐Hochberg correction method.

**TABLE S1.** Dynamics of species biomass (cells ml^−1^) and biovolume (mm^3^ L^−1^) for both Nutrient+ and Nutrient− experiments.

**TABLE S2.** The core microbiome detected in all selected samples in the Nutrient+ experiment.

## Data Availability

The data that support the findings of this study are openly available in EBI‐ENA: https://www.ebi.ac.uk/ena/browser/view/PRJEB70923.
