# Supplementary material for: Effects of salinity and nutrient stress on a toxic freshwater cyanobacterial community and its associated microbiome: An experimental study
Source: Environ Microbiol Rep. 2024 Oct 24;16(5):e70029. doi: 10.1111/1758-2229.70029 (PMC11499623; doi:10.1111/1758-2229.70029)
Supplement: Supplementary file 1 — FIGURE S1. Dynamics of the relative abundance (%) of mcyB gene over the total Microcystis population (PC gene) as a function of salinity in both Nutrient+ and Nutrient− conditions. FIGURE S2. Dynamics of intracellular and extracellular microcystins concentrations (μg L−1) together with the evolution of the different variants (%) as a function of salinity for both Nutrient+ and Nutrient− conditions. FIGURE S3. Dynamics of relative abundances of cyanobacterial sequences at the family levels in the free‐living (FREE) and attached (ATTACHED) fractions as a function of salinity under Nutrient+ (top panel) and Nutrient− (bottom panel) conditions. FIGURE S4. Alpha diversity box‐plot displaying the number of ASVs observed, Chao1, Shannon and Pielou diversity indices for the free‐living (in blue) and attached (in green) heterotrophic bacterial communities as a function of salinity for the Nutrient+ (A) and Nutrient− (B) conditions. Solid lines and asterisks indicate a significant difference. P‐values were calculated to compare alpha diversities based on a two‐sample t test using a nonparametric method with Benjamini‐Hochberg correction method. [file EMI4-16-e70029-s003.docx]

**SUPPLEMENTARY MATERIALS**


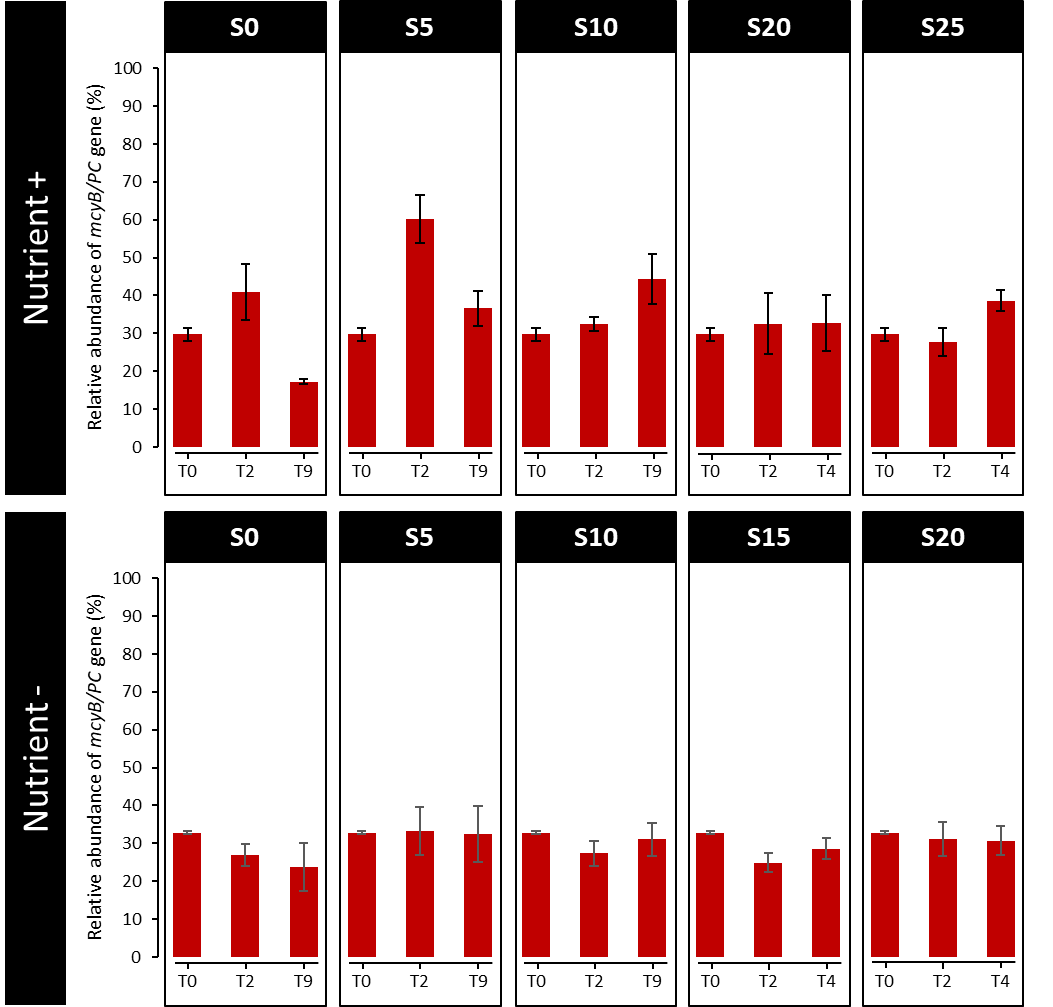


**Figure S1.** Dynamics of the relative abundance (%) of *mcyB* gene over the total *Microcystis* population (*PC* gene) as a function of salinity in both Nutrient+ and Nutrient– conditions.


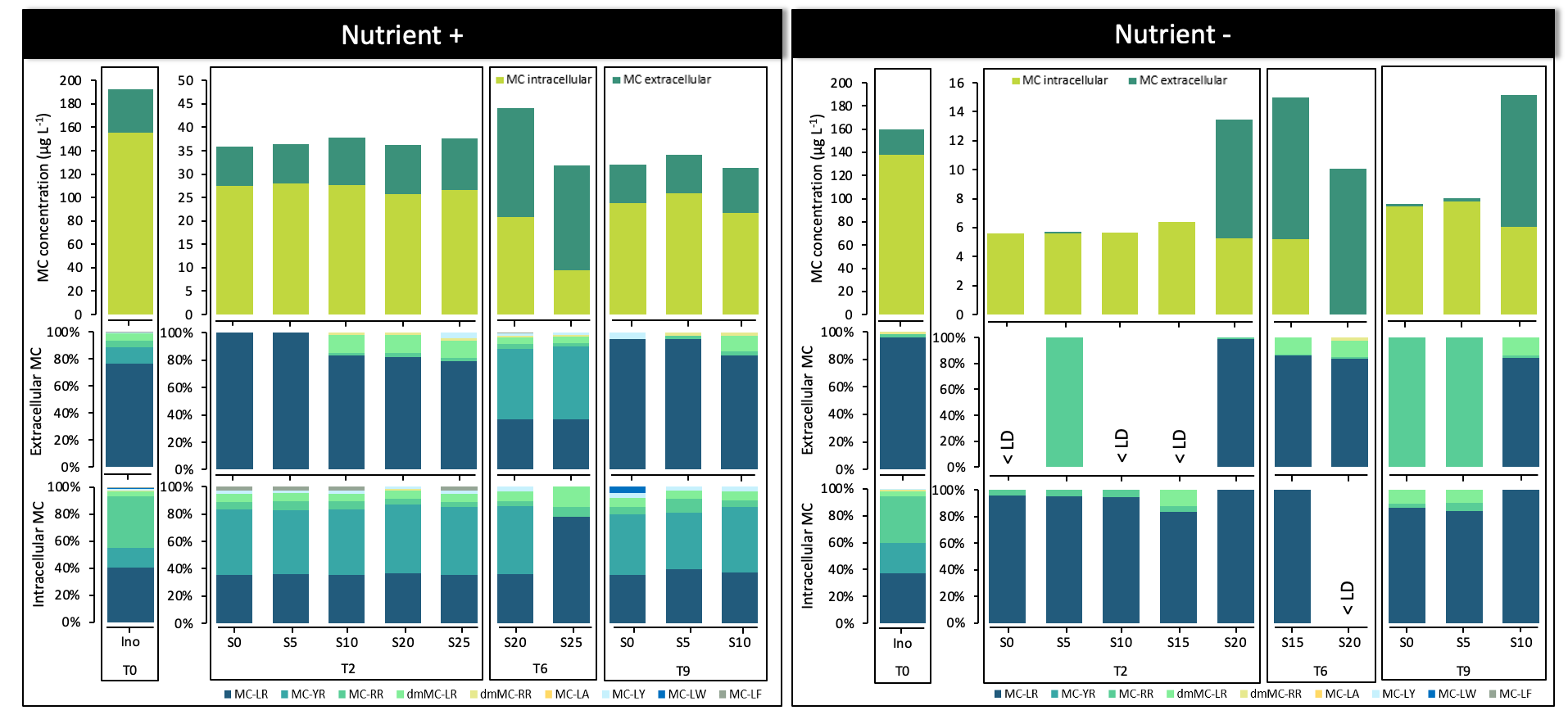


**Figure S2.** Dynamics of intracellular and extracellular microcystins concentrations (μg L^-1^) together with the evolution of the different variants (%) as a function of salinity for both Nutrient+ and Nutrient– conditions.


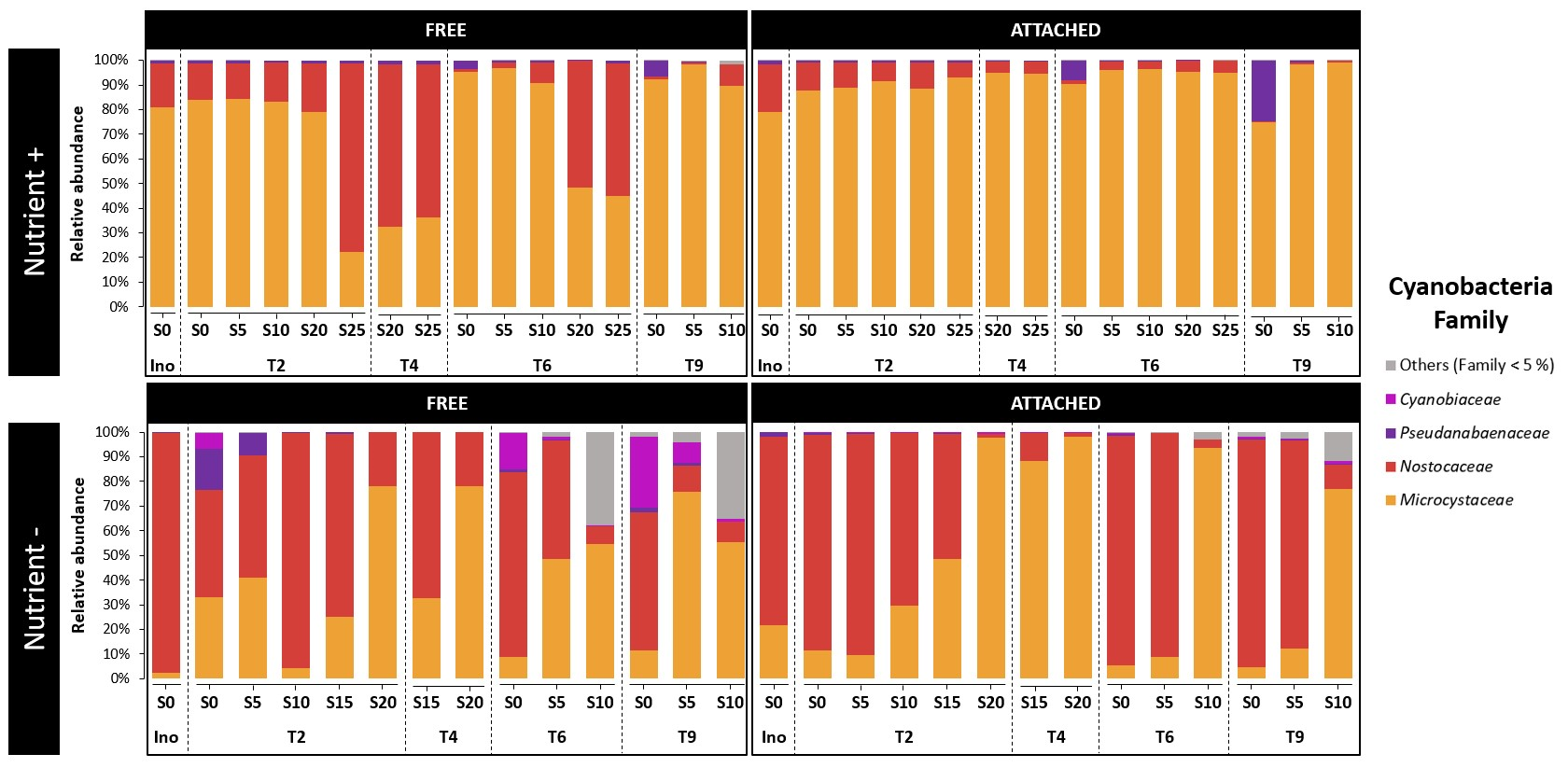


**Figure S3.** Dynamics of relative abundances of cyanobacterial sequences at the family levels in the free-living (FREE) and attached (ATTACHED) fractions as a function of salinity under Nutrient+ (top panel) and Nutrient- (bottom panel) conditions.


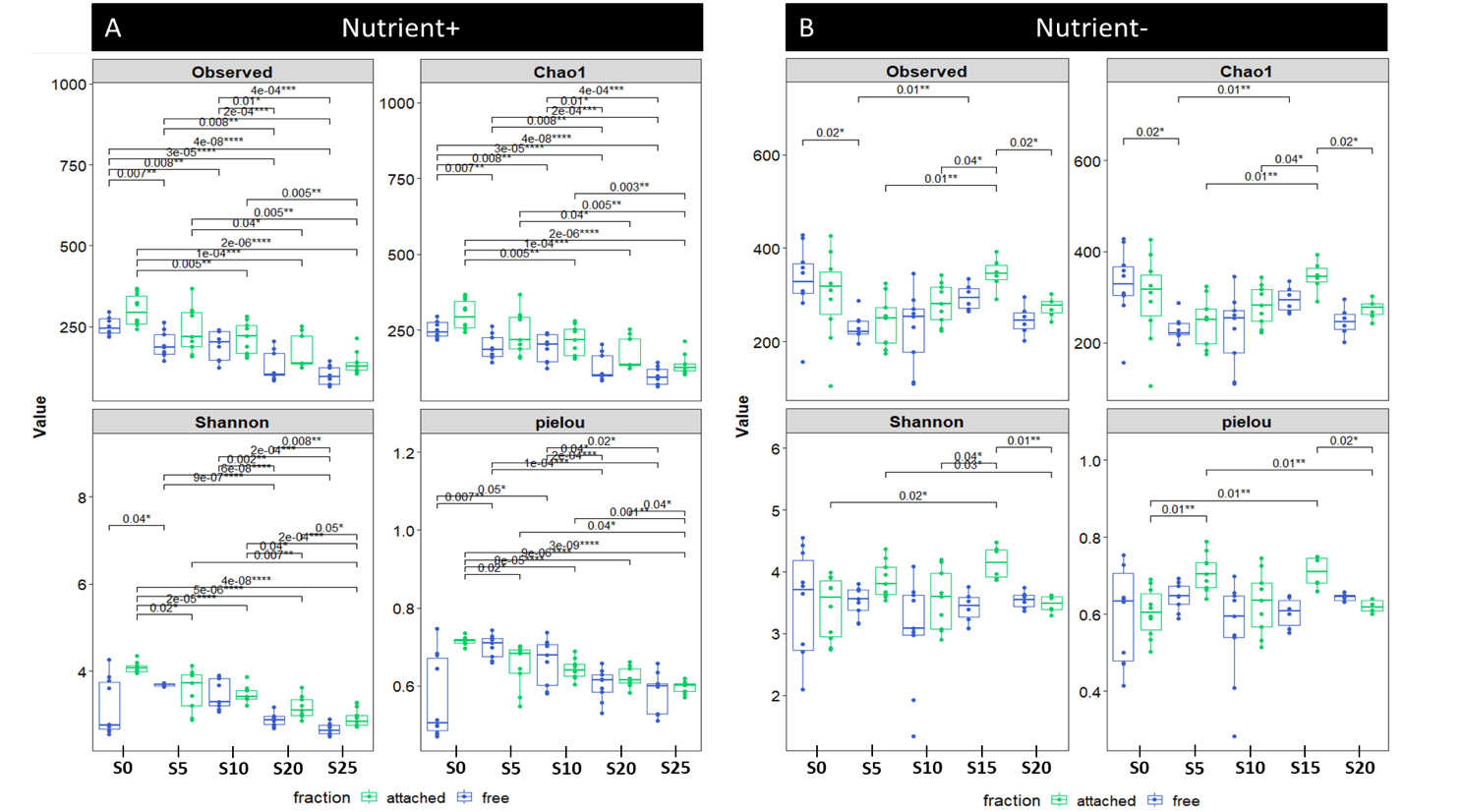


**Figure S4.** Alpha diversity box-plot displaying the number of ASVs observed, Chao1, Shannon and Pielou diversity indices for the free-living (in blue) and attached (in green) heterotrophic bacterial communities as a function of salinity for the Nutrient+ (**A**) and Nutrient- (**B**) conditions. Solid lines and asterisks indicate a significant difference. P-values were calculated to compare alpha diversities based on a two-sample t-test using a non-parametric method with the Benjamini-Hochberg correction method.
